# Hemodiafiltration May Be Associated with Senescence-Related Phenotypic Alterations of Lymphocytes, Which May Predict Mortality in Patients Undergoing Dialysis

**DOI:** 10.3390/ijms252010925

**Published:** 2024-10-11

**Authors:** Georgios Lioulios, Asimina Fylaktou, Aliki Xochelli, Theodoros Tourountzis, Michalis Christodoulou, Eleni Moysidou, Stamatia Stai, Lampros Vagiotas, Maria Stangou

**Affiliations:** 1 Department of Nephrology, 424 Military Hospital of Thessaloniki, 56429 Thessaloniki, Greece; pter43@yahoo.gr; 2 Department of Immunology, National Peripheral Histocompatibility Center, General Hospital Hippokration, 54642 Thessaloniki, Greece; fylaktoumina@gmail.com (A.F.); aliki.xochelli@gmail.com (A.X.); 3 Protypo Chronic Dialysis Unit, 55535 Thessaloniki, Greece; ttourou@gmail.com; 4 First Department of Nephrology, General Hospital Hippokration, 54642 Thessaloniki, Greece; michalischristodoulou22@gmail.com (M.C.); moysidoueleni@yahoo.com (E.M.); staimatina@yahoo.gr (S.S.); 5 School of Medicine, Aristotle University of Thessaloniki, 54642 Thessaloniki, Greece; 6 Department of Transplant Surgery, General Hospital Hippokratio, 54642 Thessaloniki, Greece; lampisv@yahoo.gr

**Keywords:** end-stage kidney disease, dialysis, hemodiafiltration, immune senescence, immune exhaustion

## Abstract

Senescence-resembling alterations on the lymphocytes of patients undergoing dialysis have been widely described. However, the pathophysiology behind these phenomena has not been clarified. In this study, we examined the impact of dialysis prescription on T and B lymphocytes, in patients undergoing dialysis.: T and B cell subsets were determined with flow cytometry in 36 patients undergoing hemodialysis and 26 patients undergoing hemodiafiltration, according to the expression of CD45RA, CCR7, CD31, CD28, CD57, and PD1 for T cells, and IgD and CD27 for B cells. The immune phenotype was associated with dialysis modality, hemofiltration volume, and mortality. Compared with hemodialysis, patients undergoing hemodiafiltration had a significantly decreased percentage of CD4+CD28-CD57- T cells [3.8 (2.4–5.3) vs. 2.1 (1.3–3.3)%, respectively, *p* = 0.002] and exhausted CD4+ T cells [14.1 (8.9–19.4) vs. 8.5 (6.8–11.7)%, respectively, *p* = 0.005]. Additionally, the hemofiltration volume was negatively correlated with CD8+ EMRA T cells (r = −0.46, *p* = 0.03). Finally, the increased exhausted CD4+ T cell percentage was associated with increased all-cause mortality in patients undergoing dialysis, independent of age. Hemodiafiltration, especially with high hemofiltration volume, may have beneficial effects on senescence-related immune phenotypes. Immune phenotypes may also be a predicting factor for mortality in patients undergoing dialysis.

## 1. Introduction

Immune alterations in end-stage kidney disease (ESKD) have been widely described and involve both innate and acquired immunity [1]. These disturbances are closely related to the persistent and chronic activation of inflammatory response, and are implicated in several clinical complications of ESKD, such as susceptibility to serious infections, inadequate response to vaccination, increased malignancy incidence, frailty, and nutritional disturbances, and, above all, increased cardiovascular risk and mortality [2,3,4,5].

In the setting of ESKD, acquired immunity alterations have been more extensively studied. Several studies show that lymphocytes from patients with ESKD exhibit phenotypic and functional characteristics of older individuals, thus resembling prematurely senescent cells. These include lymphopenia with inverted CD4+/CD8+ ratio, elimination of naïve and low differentiation subpopulations, increased cytotoxicity, decrease in regulatory T cells, and imbalance among Th responses [6,7,8].

The hallmark of senescence in T cell populations is the loss of CD28 expression on the surface of memory T cells, usually after repetitive reactivation, and the expression of the epitope CD57. These cells (CD28-CD57+) demonstrate an antigen-specific high cytotoxic capacity, as seen by the high expression of cytoplasmic granules containing cytotoxic proteins, such as perforin and granzyme-B. Moreover, they express the majority of senescence characteristics, such as a decrease in telomere length and telomerase activity, and a limited activation capacity. They also secrete a variety of proinflammatory factors known as the “senescence-associated secretory phenotype” [9] Additionally, senescent T cell subsets are characterized by a shift towards more differentiated memory populations, such as effector memory T cells re-expressing CD45RA on their surface, a molecule found mainly on the surface of naïve populations [7]. On the other hand, T cell exhaustion is a different process, during which T cells lose their effector function after continuous antigen stimulation and express inhibitory receptors such as programmed cell death protein 1 (PD1) and killer cell lectin-like receptor G 1. Despite their distinct pathophysiologic and functional differences, senescence and exhaustion may overlap [9,10].

Several pathophysiological mechanisms have been proposed to elicit these alterations. Premature thymic involution and cytokine dysregulation, particularly the decrease in interleukin 7 and increase in tumor necrosis factor α, have been associated with naïve T cell elimination and senescent CD28- T cell accumulation [11,12]. Moreover, dialysis-related factors, including dialyzer biocompatibility, water contamination, frequent catheter infection, and volume and sodium overload, appear to play a major role [13,14]. Most importantly, increased levels of uremic toxins, mainly produced by impaired intestinal flora, are poorly removed by dialysis and contribute to inflammatory activation [15].

Conventional hemodialysis (HD) is the most widespread method of kidney replacement therapy and relies on solute clearance with diffusion. However, its capacity to remove larger molecules is limited due to the relatively low speed of diffusion of these molecules through the dialyzer membrane. Online hemodiafiltration (HDF) provides an additional solute clearance with convection, which is achieved by the removal of large fluid volumes through a membrane of high hydraulic permeability (“high flux” membrane) [16,17,18,19]. The fluid removed is substituted with a sterile dialysate of optimal electrolyte composition, infused directly into the bloodstream of the patient; hence, ultra-pure water is needed [20]. Online HDF improves the removal of middle molecular weight solutes. Most importantly, it appears to have a beneficial effect on all-cause and cardiovascular mortality, oxidative stress, and hospitalization rates, and also improves patients’ quality of life [21,22]. Moreover, a reduction in inflammatory markers such as interleukin-6 and tumor necrosis factor α has been associated with HDF [23,24].

Despite the wide range of clinical associations that immune alterations have on patients with ESKD, there are no data available regarding the impact of dialysis prescription on immune phenotypes. The present study aims to investigate for potential discrepancies in lymphocytic phenotypes between patients undergoing ESKD on two main dialysis modalities (HD and HDF), examine the role of hemofiltration volume (HFV), and explore the impact of immune phenotypes on mortality.

## 2. Results

### 2.1. Patients’ Characteristics

Patient demographics are shown in Table 1. The patients were equally distributed within the three age groups. Sex distribution did not differ among the three age groups (*p* = 0.26); however, patients in the 20–40 year age group had a significantly lower body weight and body mass index (*p* = 0.03). In total, 30% of the patients had a history of immunosuppressive treatment, either due to their primary cause of ESKD, or after kidney transplantation. However, the vast majority of patients had ceased any immunosuppressive treatment for a considerable time period before study initiation (Table 1). Dialysis prescription differed among the groups, as more patients were on conventional HD in the older group 61–80 years (*p* = 0.02), without significant difference in the mean duration of each dialysis session between the groups (*p* = 0.14). Vintage of dialysis differed significantly among the groups, as patients in the middle age group 41–60 years, had a mean vintage of 101 ± 81 months, *p* = 0.01, in comparison with the young patient group (45 ± 48 months) or the older patient group (67 ± 44 months).

### 2.2. Comparison of Immune Phenotypes between Patients Undergoing Dialysis and Healthy Controls

Patients undergoing dialysis had decreased total lymphocyte proportions and counts in comparison with healthy controls (HC) (20.7 ± 6.7 vs. 29.2 ± 8.4%, *p* < 0.001, 1529 ± 500 vs. 2076 ± 561/μL, *p* < 0.001, respectively), which affected counts of both CD4+ and CD8+ subsets, without any difference in their proportions.

Within CD4+ T cell compartments, a decrease in low differentiation (naïve, recent thymic emigrants-RTE, and CD28+CD57-) subset counts was observed in the patient group, though without affecting their respective proportions. However, the proportion of the highly differentiated subset CD28-CD57- was twice as high in the patient group in comparison with patients undergoing HC, and an increase in the proportion of exhausted CD4+PD1+ T cells was observed. Within CD8+ T cell compartments, differences between the two groups were prominent only with regard to the expression of CD28, with patients having a lower proportion and count of low differentiation CD28+CD57- T cells and a higher proportion and count of CD28-CD57- T cells in comparison with patients undergoing HC.

Total B cells were severely diminished in patients with ESKD in comparison with HC [6.5 (4.5–9.1) vs. 12.8 (9.3–16) %, *p* < 0.001, and 97 (60–141) vs. 230 (157–385) cells/μL, *p* < 0.001, proportion and count, respectively), with naïve B cells being the most affected subset. Comparisons between proportions and counts of CD4+, CD8+ T cell, and B cell subsets between patients with ESKD and HC are shown in Supplemental Tables S1, S2, and S3, respectively.

To adjust for age in both patients and HC, subsets that differed between the two populations were analyzed with analysis of covariance (ANCOVA). The results are shown in Supplemental Table S4. Very briefly, differences between the patients with ESKD and HC remained significant in the multivariate model, for all the above-mentioned T and B cell subsets, with age being an independent significant factor only for low differentiation T cell subsets (CD4+ RTE, CD8+CD28+CD57-).

### 2.3. Comparison of Immune Phenotypes between Patients Undergoing HD and HDF

**Figure 1:**
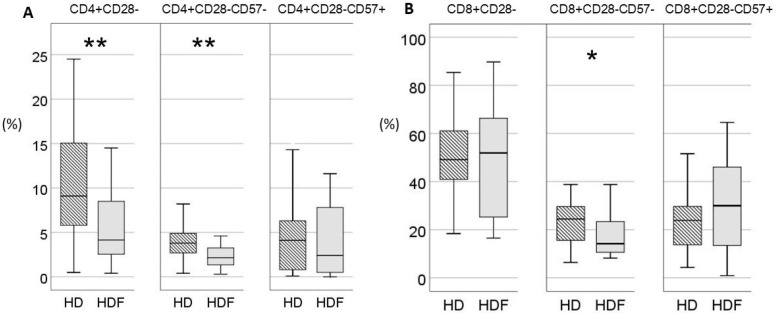
(**A**) Differences in expression of CD28 and CD57 surface markers on CD4+ T cells between patients on conventional hemodialysis (HD, N = 36, lined bars) and online hemodiafiltration (HDF, N = 26, gray bars). (**B**) Differences in expression of CD28 and CD57 surface markers on CD8+ T cells between patients on HD and HDF. T cell subsets are given as percentages. Statistic significance tested with Mann–Whitney U test, * *p* < 0.05, ** *p* < 0.01.

Online HDF was applied to 26/62 (42%) patients. The age of the patients did not differ significantly between the two groups [57 (38–71) vs. 49 (31–58) years for patients undergoing HD and HDF, respectively, *p* = 0.08]. Patients on HDF had significantly higher proportions of low differentiation CD4+CD28+CD57- and decreased proportions of highly differentiated CD4+CD28- lymphocytes. However, within the compartment of CD28- T cells, the difference between the two patient groups was restrained only in the CD28-CD57- subset, which was increased in patients undergoing HD, while terminally differentiated CD28-CD57+ T cells did not differ between the two groups (Figure 1). In the CD8+ compartment, proportions of CD28+CD57- T cells and total CD28- T cells did not differ between the two subgroups, whereas CD8+CD28-CD57- T cells were significantly reduced in the HDF group. Absolute numbers and proportions of T cells according to expressions of CD28 and CD57 are shown in Supplemental Table S5.

**Figure 2:**
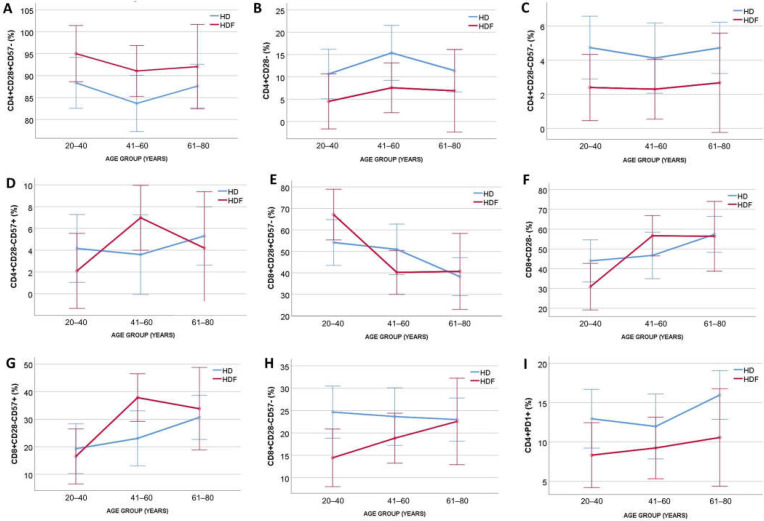
ANCOVA for modality [Hemodialysis (HD) N = 36, Hemodiafiltration (HDF) N = 26] and age group (age group 20–40 N = 21, 41–60 N-21, 61–80 N = 20) for different T cell subset percentages. (**A**) Low differentiated CD4+CD28+CD57- T cells. (**B**) Highly differentiated CD4+CD28- T cells. (**C**) Highly differentiated CD4+CD28-CD57- T cells. (**D**) Senescent CD4+CD28-CD57+ T cells. (**E**) Low differentiated CD8+CD28+CD57- T cells. (**F**) Highly differentiated CD8+CD28- T cells. (**G**) Highly differentiated CD8+CD28-CD57- T cells. (**H**) Senescent CD8+CD28-CD57+ T cells. (**I**) Exhausted CD4+ T cells. The blue line represents patients undergoing HD, the red line represents patients undergoing HDF.

To adjust for age, the two patient cohorts were divided into three age groups and ANCOVA was performed. Patients on HDF had increased proportions of CD4+CD28+CD57- (*p* = 0.03, for modality), and decreased proportions of CD4+CD28- (*p* = 0.02, for modality) and CD4+CD28-CD57- (*p* = 0.02, for modality), independent of age (*p* = 0.37, *p* = 0.41, and *p* = 0.88 for the three subsets, respectively), with no significant interaction between age group and modality (*p* = 0.9, *p* = 0.88, and *p* = 0.9, respectively). Proportions of CD4+CD28-CD57+ T cells remained non-significant for dialysis modality and age group (Figure 2). In contrast, CD8+ subsets were affected by age in ANCOVA (*p* = 0.003, *p* = 0.006, and *p* = 0.01 for CD28+CD57-, CD28-, and CD28-CD57-, respectively), while neither dialysis modality nor age were significant in ANCOVA for CD8+CD28-CD57- T cell proportions (*p* = 0.06, *p* = 0.61, and *p* = 0.358 for modality, age, and interaction, respectively) (Figure 2).

Moreover, there was a significant difference in percentage of exhausted CD4+PD1+ cells [14.1 (8.9–19.4) vs. 8.5 (6.8–11.7)% for patients undergoing HD and HDF, respectively, *p* = 0.005], whereas the percentage of CD8+PD1+ cells did not differ significantly between the two groups [33.8 (12.7–52.2) vs. 17.4 (8.5–41)% for patients undergoing HD and HDF, respectively, *p* = 0.06]. The impact of age in ANCOVA was not significant in the CD4+PD1+ (*p* = 0.42) or the CD8+PD1+ T cell percentages (*p* = 0.82). The significance of dialysis modality was retained after adjusting for age only for CD4+PD1+ T cell percentages (Figure 2).

Counts and percentages of naïve and memory T and B cells did not differ between the two groups. Moreover, levels of urea, creatinine, and phosphorous levels did not correlate with any of the studied T and B cells subsets 

### 2.4. Effect of HFV

**Figure 3:**
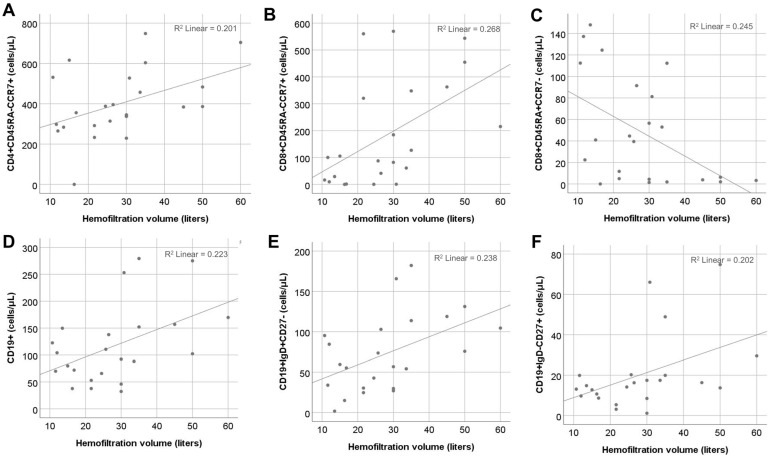
Spearman correlation of hemofiltration volume applied to patients undergoing HDF (N = 26) with different T cell and B cell subsets. (**A**) Central memory CD4+ T cells. (**B**) Central memory CD8+ T cells. (**C**) Effector memory CD8+ T cells re-expressing CD45RA (EMRA). (**D**) Total B cells. (**E**) Naïve B cells. (**F**) Switched memory B cells.

The correlation of T cell subsets with hemofiltration volume in patients on HDF revealed a significant positive association between HFV and central memory CD4+ (r = 0.46, *p* = 0.03) and CD8+ cell counts (r = 0.51, *p* = 0.01) and a significant negative correlation with CD8+ effector memory T cells re-expressing the CD45RA (EMRA) count (r = −0.46, *p* = 0.03). Moreover, a positive correlation between HFV with the total B cell count was observed (r = 0.46, *p* = 0.03). Out of the B cell subpopulations, HFV mainly affected naïve (r = 0.53, *p* = 0.008) and switched memory B cells (r = 0.5, *p* = 0.02), whereas its association with IgD-CD27- B cells (r = 0.41, *p* = 0.05) was marginal. (Figure 3). In the univariate regression model, the effect of HFV remained significant for all the above subsets, except for IgD-CD27- B cells (Table 2).

Multivariate analysis performed for each lymphocyte population, including age and HFV, showed that HFV was an independent factor for CD8+ CM and EMRA T cell population, whereas age was the main factor affecting CM CD4+, total B, and naïve B cell counts (Table 2).

### 2.5. Effect of Vintage

**Figure 4:**
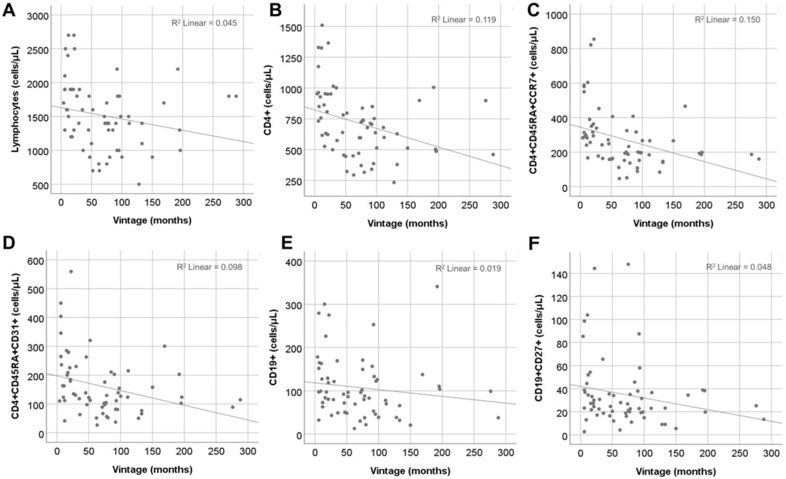
Spearman correlation of dialysis vintage of all patients (N = 62) with different T cell and B cell subsets. (**A**) Total lymphocytes. (**B**) Total CD4+ T cells. (**C**) Naïve CD4+ T cells. (**D**) CD4+ recent thymic emigrants. (**E**) Total B cells. (**F**) Memory B cells.

Dialysis vintage was negatively correlated with the total count of T cells and CD4+ T cells (r = −0.35, *p* = 0.005 and r = −0.46, *p* < 0.001, respectively), while total CD8+ T cells were not affected. The effect of vintage was evident only in low differentiation CD4+ T cell subsets (r = −0.51, *p* < 0.001, r = −0.38, *p* = 0.002, and r = −0.31, *p* = 0.01 for naïve, RTE, and central memory (CM) CD4+ T cells, respectively). Moreover, there was a significant negative correlation with CM CD8+ T cells (r = −0.29, *p* = 0.02). Finally, total B cell and memory B cell counts were also negatively correlated with dialysis vintage (r = −0.26, *p* = 0.04 and r = −0.25, *p* = 0.04) (Figure 4). However, in multivariate analysis for vintage and age, the effect of vintage remained significant only for total CD4+ and naïve CD4+ counts, whereas age was the main predictive factor for CD4+ RTEs and B cell subsets (Table 3). Total lymphocytes and CM cells, in both CD4+ and CD8+ compartments, were not affected by vintage or age in the multivariate model.

### 2.6. Immune Phenotype May Be Related to Increased Mortality

**Figure 5:**
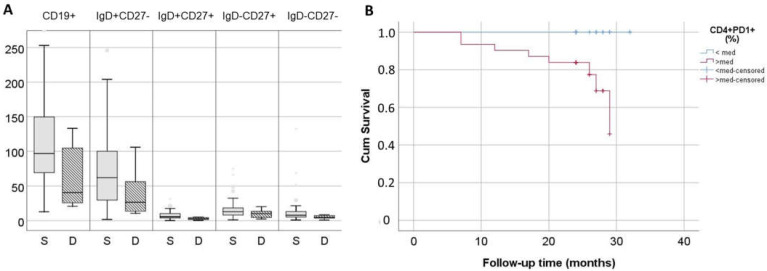
(**A**) Comparison of B cell subsets between survivors-gray bar (N = 54) and deceased patients-lined bar (N = 8), statistic significance tested with Mann–Whitney U test. (**B**) Kaplan–Meier survival analysis of patients undergoing dialysis according to the percentage of exhausted CD4+ T cells (N = 32 with CD4+PD1+ > median = 11.7%, red line and N = 32 with CD4+PD1+ < median, blue line). S—survivors, D—deceased.

All patients and healthy controls were followed up prospectively after phenotypic evaluation for at least 24 months and deaths were recorded. In total, 13% (8/62) of patients died during the follow-up period. No deaths occurred in the healthy control group. Patients undergoing dialysis had an increased proportion of exhausted CD4+PD1+ T cells in comparison with healthy controls [11.6 (7–17.8) vs. 7.8 (5.4–11.7)% for patients and healthy controls, respectively, *p* = 0.01], with no difference in the corresponding CD8+ T cell subset. The percentage of CD4+PD1+ T cells had a tendency to increase with age, but did not reach statistical significance in comparison with healthy controls, the percentage of whom did differ among the three age groups (Supplemental Figure S1). Deceased patients had significantly higher proportions of exhausted CD4+PD1+ T cells in comparison with survivors [18.8 (14–25) vs. 11.5 (7–16.9)%, respectively, *p* = 0.006] and decreased counts of total B cells [40 (24–115) vs. 97 (70–150)cells/μL, respectively, *p* = 0.02]. This difference affected mainly naïve and IgM memory B cells (Figure 5A). Kaplan–Meier survival analysis for patients with proportions of CD4+PD1+ above and below median was significant (*p* = 0.002) (Figure 5B). Moreover, a significant effect of exhausted CD4+PD1+ T cell proportion remained in Cox regression, either when examined alone as a continuous variable (*p* = 0.003, 95%CI: 1.0, 1.2) or in combination with age (CD4+PD1+ proportion: *p* = 0.03, 95% CI: 1.01, 1.23, age: *p* = 0.02, 95% CI: 1.01, 1.44).

## 3. Discussion

Alterations of acquired immunity have been studied to a certain extend in ESKD. However, the effect of dialysis modality and prescription has not been studied to date. The scope of the present study was to examine differences of senescence-related phenotypes of T and B lymphocytes between patients on conventional HD or online HDF, to determine the effect of hemofiltration volume and dialysis vintage, and to investigate the impact of T and B cell subsets on mortality.

Dialysis modality appeared to have a significant effect on T cell phenotype, in terms of the expression of CD28 and PD1 molecules on CD4+ and CD8+ T lymphocytes. Patients on HDF had an improved phenotype with a higher percentage of low differentiation CD28+ and a lower percentage of senescent CD28-CD57- T cells. CD28, a costimulatory molecule, is downregulated after multiple activations of a T cell clone [25], or as a result of cytokine action in the absence of antigenic stimulation [26,27,28], and has been documented in many chronic inflammatory conditions, including ESKD [8,29,30]. High percentages of CD4+CD28- T cells have been associated with instable angina and the increased risk of relapse of an acute coronary event or stroke [31,32,33,34,35]. Moreover, these cells have been found within atheromatous plaque in animals and have been associated with vascular smooth muscle cell apoptosis and an increased risk of plaque rupture [36] CD8+CD28- T cells were also found to be increased in patients with cardiovascular disease undergoing dialysis [37]. Data on the role of CD4+PD1+ T cells on patient outcome are inadequate, as discussed later. However, a study by Lorenz et al. reports a reduction in CD4+PD1+ T cells in patients undergoing dialysis with medium cut-off dialyzers, which, in accordance with our results, may indicate a potential role of dialysis modality [38].

Significant correlations between T cell phenotype with the hemofiltration volume were found in patients undergoing HDF. Our analysis revealed a clear positive effect of increased hemofiltration volume on central memory T cells, both in CD4+ and CD8+ compartments, while hemofiltration volume was negatively correlated with CD8+ EMRA T cells, a terminally differentiated subset with strong senescent characteristics [35]. Interestingly, CD8+ EMRA cell population has been positively associated with a protein-bound uremic toxin, p-cresyl sulfate [39]. Hence, an increased clearance of protein-bound uremic toxins, potentially achieved with HDF, may contribute to the improvement of the immune phenotype of dialysis patients. Finally, a potential positive effect was also found in B cell compartments; however, in multivariate analysis, age was the main determinant of B cell subsets.

Dialysis vintage was also associated with several immunophenotype alterations. Vintage was the main determinant of total CD4+ and naïve CD4+ T cells, whereas age was the main determinant of CD4+ RTE and B cells in the multivariate model, despite vintage being significant in the linear correlation analysis. In 2011, Borges et al. reported a positive association between CD4+ apoptotic cells with dialysis vintage, a finding consistent with the results of the present study [40]. Central memory T cell counts had a significant negative correlation with vintage, which, however, was not retained in the multivariate analysis. Results from previous studies are controversial, as a recent large multicenter study supported the effect of dialysis vintage on the percentage of central memory CD4+ cells [39], while other investigators claimed no significant correlation [29]. Consistent with our results, Stefanidis et al. have recently reported a gradual reduction in T lymphocyte telomere length during dialysis vintage [41].

Finally, according to the findings of the present study, immune phenotypes might predict all-cause mortality in patients undergoing dialysis. The literature on the role of immune phenotypes on the survival of patients undergoing is, to date, poor. One study, published in 2020, associated decreased levels of low-differentiation T cell subsets with increased all-cause mortality in patients undergoing dialysis [42]. These findings were not confirmed by the present study, potentially due to the lower number of patients and a shorter follow-up time. B cell counts < 100/μL were also associated with increased cardiovascular and all-cause mortality in patients undergoing dialysis, according to a study by Molina et al. [43].

The present study adds one more potential predictive factor associated with mortality in this patient group: the percentage of exhausted CD4+ T cells, defined by the expression of inhibitory receptor PD1. Exhausted T cells have been associated with inadequate antigen clearance in viral infections and drug-resistant tumors, but this state appears to be reversible with PD1 blockade and the cells being able to retrieve their effector functions. Data on T cell exhaustion in patients with ESKD are scarce. A study published in 2020 reports significantly higher levels of exhausted CD4 T cells in patients with ESKD and patients undergoing dialysis, speculating that this alteration derives from chronic antigen stimulation potentially due to the toxic action of uremic toxins. However, no clinical significance is reported [44]. Of note, increased percentages of exhausted CD4+ T cells have been associated with better renal graft function six months post kidney transplantation [45].

A major limitation of the present study is the inadequate definition of senescent and exhausted cellular subsets. The surface phenotype used has been largely associated with several senescence-related T cell disturbances, such as increased concentration of senescence-associated beta-galactosidase, decreased telomere length and telomerase activity, and the so-called senescence-associated secretory phenotype, in different patient groups. However, the main purpose of our study was to investigate, among easily assessed markers, the most suitable(s) for potential mortality biomarkers in this specific patient population. We believe that, after our results, it would be justified to focus, for example, on the potential role of PD1+ in the survival of patients undergoing dialysis, and to study deeper this specific subset. Other limitations include the relatively low number of patients enrolled and the limited follow-up interval. Moreover, patients were not initiated in a randomized way in HD of HDF before the initiation of this study. Thus, more studies are needed to confirm our results.

In conclusion, HDF is potentially beneficial to patients as it is associated with improved immune phenotypes in terms of CD28 and PD1 expression on T cells. An additional benefit was observed in high hemofiltration volumes, which were associated with a decreased count of terminally differentiated CD8+ EMRA T cells. Moreover, dialysis vintage affected total and naïve CD4+ T cell counts, the latter, however, being an unmodifiable factor. Finally, exhausted CD4+ T cells might be a predictive marker for all-cause mortality in patients undergoing dialysis.

## 4. Materials and Methods

### 4.1. Patients

In this prospective observational study, a total of 62 adult patients on chronic maintenance HD were included and followed up for at least two years. All the patients received a thrice-weekly dialysis program with HD or online HDF for at least one year, with Kt/V > 1.2. Patients with active malignancy, hematological or autoimmune diseases, recent infection, or vaccination (<3 months), or patients who had received immunosuppressive treatment one year before enrollment were excluded from the study. As diabetes mellitus has been shown to provoke alterations to cellular immunity, patients with diabetes were deemed not eligible for this study [46,47]. Moreover, patients who changed from HD to HDF or vice versa, for any reason, were also excluded. Because many lymphocyte subpopulations are affected by ageing, though not in a linear way, we decided to stratify patients in three age groups: 20–40, 41–60, and 61–80 years. Additionally, 34 age- and sex-matched healthy volunteers were included as a control group. Patients and healthy controls were followed up for two years and cardiovascular events, de novo cancer diagnoses, and deaths were recorded. This study was approved by the Institutional Review Board of the Medical School of the Aristotle University of Thessaloniki (ref No. 2273/15-12-2020) and was conducted according to the principles of the Declaration of Helsinki. All study participants signed an informed consent prior to enrollment.

### 4.2. Flow Cytometry

Flow cytometry was performed in fresh total blood, collected immediately before the initiation of a mid-week dialysis session, processed no more than 12 h after collection. Specimens were kept in the refrigerator at 4 °C until examination. Proportions of CD4+, CD8+, and B lymphocytes subsets were determined using a cell counter (Navios Flow Cytometer, Beckman Coulter), according to the manufacturer’s recommendations, as described previously [48]. For each sample, four different panels of markers were prepared. Briefly, the lymphocytes were stained with conjugated antibodies as following: anti-CD45 PC7 J33 (IM3548U, Beckman Coulter), anti-CD3 FITC UCHT1 (A07746, Beckman Coulter), anti-CD3 PE UCHT1 (A07747, Beckman Coulter), anti-CD4 Pacific blue MEM-241 (PB-359-T100, EXBIO, Praha SA), anti-CD8 PC5 B9.11 (A7758, Beckman Coulter), anti-CD45RA APC MEM-56 (1A-223-T100, EXBIO, Praha SA), anti-CCR7 PE 4B12 (1P-735-C100, EXBIO, Praha SA), anti-CD28 CD28.2 PE-EF610 (61-0289-42, ThermoScientific LSG), anti-CD31 APC MEM-05 (T5-273-T100, EXBIO, Praha SA), anti-CD57 FITC TB01 (1F-158-T100, EXBIO, Praha SA), and anti-CD279 (PD1) EI12.2H7 (11-176-C100, EXBIO, Praha SA)., anti-CD19 PC5 (J3-119, Beckman Coulter), anti-CD27 ECD (1A4CD27, Beckman Coulter), and anti-IgD (IA6-2, EXBIO, Praha SA), in four different tubes, as described in Supplemental Table S6, and lymphocyte subsets were defined, as shown in Supplemental Table S7. The gating strategy is shown in Supplemental Figure S2.

### 4.3. Statistical Analysis

Statistical analysis was performed with SPSS.25 for Windows (IBM, Armonk, NY, USA). Continuous variables were reported as median (25th–75th percentile). Differences between groups were evaluated using Chi-square test for categorical variables. Comparisons of continuous variables were evaluated with Mann–Whitney U test for comparison between two groups or Kruskal–Wallis test for comparisons between three groups. Analysis of covariance (ANCOVA) was used to evaluate the effect of age in comparisons between subgroups. Spearman rank correlation coefficient was used to estimate correlation between continuous variables. Multivariate linear regression was used for the significance of multiple continuous variables. For clinical outcomes of patients, Kaplan–Meier and Cox regression model analyses were performed. A *p*-value < 0.05 was considered statistically significant.

## Figures and Tables

**Table 1:** Patients’ baseline characteristics.

Age Group	20–40	41–60	61–80	Total
Ν	21	21	20	62
Mean age ± sd, years	30.5 ± 6.3	52.2 ± 5.6	71.2 ± 6.4	51.3 ± 17.7
Sex, male/female	14/7	14/7	9/11	37/25
Mean weight ± sd, kg	61 ± 11.5	81.3 ± 19.2	71.1 ± 12.5	71.3 ± 17
Mean BMI ± sd, kg/m^2^	21 ± 3.2	25.9 ± 3.9	25.5 ± 3.6	24.5 ± 4.1
Cause of ESKD				
Primary glomerulonephritis (%)	3 (14.3)	10 (47.6)	4 (20)	17 (27.4)
Hypertension	1 (4.8)	2 (9.5)	0 (0)	3 (4.8)
Obstructive nephropathy	5 (23.8)	1 (4.8)	2 (10)	8 (12.9)
Polycystic kidney disease	1 (4.8)	2 (9.5)	4 (20)	7 (11.2)
Other	6 (28.6)	1 (4.8)	1 (5)	7 (11.2)
Unknown	5 (23.8)	5 (23.8)	9 (45)	19 (30.6)
Dialysis characteristics				
Classic hemodialysis	11	9	16	36
Online hemodiafiltration	10	12	4	26
Mean vintage ± sd, months	45 ± 48	101 ± 81	67 ± 44	72 ± 64
Kt/V ± sd	1.29 ± 0.05	1.27 ± 0.06	1.23 ± 0.04	1.27 ± 0.07
Mean dialysis duration ± sd, Min	240 ± 14	246 ± 11	234 ± 13	240 ± 14
Comorbidities				
Hypertension	11	11	10	32
Cardiovascular disease	2	11	8	21
Secondary hyperparathyroidism	11	12	11	34
Anemia	18	15	18	51
History of kidney transplantation (%)	4 (19)	6 (28.6)	0 (0)	10 (16.1)
Prior immunosuppression (%)	5 (23.8)	11 (52.3)	3 (15)	19 (30)
Median time since immunosuppression cessation (IQR), months	75 (43–148)	45 (36–63)	48 (-)	48 (40–96)
Laboratory values, median (IQR)				
Hemoglobin, g/dL	11.6 (10.8–11.9)	11.2 (10.4–11.7)	10.5 (10–11.1)	11 (10.5–11.6)
Urea, mg/dL	130 (96–149)	145 (117–154)	121 (112–140)	127 (110–149)
Creatinine, mg/dL	9.7 (7.8–11.8)	9.1 (7.1–10.9)	8.4 (5.8–9.5)	9.1 (7.1–10.8)
Calcium, mg/dL	9.1 (8.8–9.2)	8.9 (8.8–9.5)	9.2 (8.7–9.6)	9.1 (8.8–9.3)
Phosphorus, mg/dL	4.6 (4–5.5)	4.6 (3.3–5)	4 (3.8–4.5)	4.4 (3.8–5.1)
Parathormone, pg/mL	162 (89–391)	268 (145–376)	197 (98–244)	206 (106–353)
Albumin, g/dL	4.3 (3.9–4.4)	4.2 (3.9–4.3)	3.9 (3.8–4.1)	4.1 (3.9–4.3)
C reactive protein (mg/L)	1.7 (1.2–3.7)	2.5 (1.4–7.4)	3.7 (1.8–7.4)	2.4 (1.4–4.7)

**Table 2:** Univariate linear regression of T and B cell subsets for hemofiltration volume and multivariate regression of T and B cell subsets for hemofiltration volume and age.

Lymphocyte Subsets (Cells/μL)	Univariate Linear Regression				Multivariate Linear Regression					
	Hemofiltration Volume				Hemofiltration Volume			Age (Years)		
	B	R^2^	95% CI	*p*	PC	95% CI	*p*	PC	95% CI	*p*
CD4+CD45RA-CCR7+	0.45	0.2	0.5, 10.8	0.03	0.33	−1.1, 9.0	0.12	−0.43	−9.8, −0.2	0.04
CD8+CD45RA-CCR7+	0.52	0.26	1.8, 13.1	0.01	0.48	1.1, 13.3	0.02	−0.05	−6, 5	0.81
CD8+CD45RA+CCR7-	−0.49	0.24	−3.2, −0.3	0.02	−0.56	−3.6, −0.6	0.006	−0.32	−2.4, 0.3	0.13
CD19+	0.47	0.22	0.3, 4.5	0.02	0.36	−0.3, 3.8	0.09	−0.42	−3.9, −0.03	0.04
CD19+IgD+CD27-	0.48	0.23	0.3, 3.2	0.02	0.38	−0.2, 2.6	0.08	0.48	−2.8, −0.2	0.02
CD19+IgD-CD27+	0.45	0.20	−0.05, 1.1	0.03	0.39	0.05, 1.1	0.07	−0.16	−0.7, 0.35	0.47
CD19+IgD-CD27-	0.12	0.01	−0.3, 1.3	0.30	−0.07	−1.1, 0.8	0.75	−0.11	−1.2, 0.6	0.61

**Table 3:** Univariate linear regression of T and B cell subsets for dialysis vintage and multivariate regression of T and B cell subsets for dialysis vintage and age.

Lymphocyte Subsets (Cells/μL)	Univariate Linear Regression				Multivariate Linear Regression					
	Vintage (Months)				Vintage (Months)			Age (Years)		
	BC	R^2^	95% CI	*p*	PC	95% CI	*p*	PC	95% CI	*p*
Total Lymphs	−0.21	0.04	−3.6, 0.3	0.09	−0.17	−3.3, 0.6	0.18	−0.16	−12.0, 2.5	0.19
CD4+	−0.34	0.12	−2.6, −0.4	0.006	−0.30	−2.3, −0.2	0.02	−0.22	−7.1, 0.4	0.08
CD4+CD45RA+CD31+	−0.31	0.98	−0.9, −0.1	0.01	−0.23	−0.7, 0.02	0.06	−0.46	−3.9, −1.3	<0.001
CD4+CD45RA+CCR7+	−0.38	0.15	−1.6, −0.3	0.002	−0.35	−1.5, −0.2	0.006	−0.17	−3.8, 0.8	0.19
CD4+CD45RA-CCR7+	−0.17	0.03	−1.2, 0.2	0.17	−0.14	−1.2, 0.3	0.26	−0.11	−4.0, 1.5	0.37
CD8+CD45RA-CCR7+	−0.13	0.02	−1.2, −0.4	0.32	−0.08	−1.0, 0.5	0.53	−0.20	−5.3, 0.6	0.12
CD19+	−0.14	0.02	−0.4, 0.1	0.28	−0.01	−0.2, 0.2	0.88	−0.52	−3.0, −1.2	<0.001
CD19+CD27+	−0.22	0.05	−0.2, 0.02	0.08	−0.15	−0.1, 0.04	0.22	−0.29	−0.8, −0.06	0.02

## Data Availability

Data will be available from the authors upon request.

## Supplementary Materials

The following supporting information can be downloaded at: https://www.mdpi.com/article/10.3390/ijms252010925/s1.
