# Supplementary material for: Hemodiafiltration May Be Associated with Senescence-Related Phenotypic Alterations of Lymphocytes, Which May Predict Mortality in Patients Undergoing Dialysis
Source: Int J Mol Sci. 2024 Oct 11;25(20):10925. doi: 10.3390/ijms252010925 (PMC11507245; doi:10.3390/ijms252010925)
Supplement: Supplementary file 1 [file ijms-25-10925-s001.zip › ijms-3152626-supplementary.pdf]

Supplemental Table 1. Comparison of CD4+ T cell phenotype between End-stage kidney disease (ESKD) patients and healthy controls (HC)

	ESKD	HC	p
**n**	**62**	**34**	
CD4 T cell subsets (%)			
**CD4+**	47(42.4-53)	50.8(45.4-55.7)	0.05
**CD4+CD45RA+CCR7+**	35.7(29.9-44.5)	34(29.6-47.2)	0.914
**CD4+CD45RA-CCR7+**	59.9(49-66.1)	56.7(45.8-66.1)	0.44
**CD4+CD45RA-CCR7-**	0.65(0.3-1.47)	0.75(0.1-1.42)	0.456
**CD4+CD45RA+CCR7-**	1.85(0.85-3.1)	1.35(0.4-3.12)	0.381
**CD4+CD45RA+CD31+**	21.6(13.9-29.7)	26.6(16.1-31.9)	0.234
**CD4+CD28+CD57-**	92.3(84.5-95.9)	95.1(87.6-96.6)	0.083
**CD4+CD28-CD57-**	3.2(1.6-4.5)	1.5(0.7-3.7)	0.013
**CD4+CD28-CD57+**	2.8(0.7-7.1)	1.9(0.4-3.8)	0.05
**CD4+PD1+**	11.6(7-17.8)	7.8(5.4-11.7)	0.01
CD4 T cell subsets (cells/µl)			
**CD4+**	709(502-906)	992(793-1236)	<0.001
**CD4+CD45RA+CCR7+**	237(161-316)	339(221-528)	0.005
**CD4+CD45RA-CCR7+**	369(268-532)	565(338-670)	0.017
**CD4+CD45RA-CCR7-**	4(2-10)	7(1-14)	0.567
**CD4+CD45RA+CCR7-**	12(5-22)	12(3-32)	0.842
**CD4+CD45RA+CD31+**	129(98-210)	253(139-352)	<0.001
**CD4+CD28+CD57-**	614(446-783)	961(745-1166)	<0.001
**CD4+CD28-CD57-**	20(13-30)	13(8-42)	0.739
**CD4+CD28-CD57+**	20(5-47)	18(3-44)	0.706
**CD4+PD1+**	78(45-124)	69(52-119)	0.854

Supplemental Table 2. Comparison of CD8+ T cell phenotype between End-stage kidney disease (ESKD) patients and healthy controls (HC)

	ESKD	HC	p
**n**	**62**	**34**	
CD8 T cell subsets (%)			
**CD8+**	26.9(18.3-33.6)	21.8(17.2-31.4)	0.267
**CD8+CD45RA+CCR7+**	25(10.6-46.2)	21.9(9.1-38.9)	0.55
**CD8+CD45RA-CCR7+**	45.4(17.7-80.2)	34.7(6.8-77.3)	0.388
**CD8+CD45RA-CCR7-**	4(1.6-10.1)	6(1.6-13.2)	0.886
**CD8+CD45RA+CCR7-**	12.2(0.9-24.5)	10.1(2.9-30.5)	0.635
**CD8+CD45RA+CD31+**	30.1(13-47.8)	33.8(10.2-49.7)	0.771
**CD8+CD28+CD57-**	46.7(31.7-66.6)	58.3(47.9-71.3)	0.009
**CD8+CD28-CD57-**	19.8(12.5-29.1)	10.3(6.6-14.9)	<0.001
**CD8+CD28-CD57+**	26.3(13.6-40.4)	23.3(12.3-33.9)	0.360
**CD8+PD1+**	30.3(11.3-46.2)	28.8(17.6-45.4)	0.505
CD8 T cell subsets (cells/μl)			
**CD8+**	361(259-500)	459(328-755)	0.02
**CD8+CD45RA+CCR7+**	89(35-169)	127(23-198)	0.818
**CD8+CD45RA-CCR7+**	124(46-323)	137(24-284)	0.610
**CD8+CD45RA-CCR7-**	13(5-31)	22(1-78)	0.447
**CD8+CD45RA+CCR7-**	24(4-82)	47(5-147)	0.278
**CD8+CD45RA+CD31+**	109(45-180)	180(40-296)	0.139
**CD8+CD28+CD57-**	155(111-204)	286(192-374)	<0.001
**CD8+CD28-CD57-**	68(39-100)	47(29-77)	0.03
**CD8+CD28-CD57+**	89(33-189)	78(31-247)	0.7
**CD8+PD1+**	74(43-173)	120(80-200)	0.101

Supplemental Table 3. Comparison of B cell phenotype between End-stage kidney disease (ESKD) patients and healthy controls (HC)

	**ESKD**	**HC**	**p**
**n**	**62**	**34**	
B cell subsets (%)			
**CD19+**	6.5(4.5-9.1)	12.8(9.3-16)	<0.001
**CD19+IGD+CD27+**	5.8(3.5-9)	8.9(4.5-12.7)	0.032
**CD19+IGD+CD27-**	67.7(58.5-76.2)	65.4(47.5-72)	0.063
**CD19+IGD-CD27+**	15.1(10.2-18.9)	15.9(10.9-24.8)	0.27
**CD19+IGD-CD27-**	7.4(5.3-12.9)	8.1(6.3-14.6)	0.471
B cell subsets (cells/µl)			
**CD19+**	97(60-141)	230(157-385)	<0.001
**CD19+IGD+CD27+**	5(3-10)	23(11-31)	<0.001
**CD19+IGD+CD27-**	59(28-101)	130(89-248)	<0.001
**CD19+IGD-CD27+**	12(7-17)	38(23-60)	<0.001
**CD19+IGD-CD27-**	7(5-12)	21(13-38)	<0.001

Supplemental Table 4. ANCOVA for the effect of end-stage kidney disease (ESKD) and age on the proportions and absolute counts of lymphocyte populations. In the first column is presented the statistical significance of presence of ESKD, in the second column the significance of age group and in the third column the significance of both. Numbers represent the corresponding p-value after two-way ANCOVA.

	**ESKD**	**Age**	**ESKD x Age**
Total Lymphocytes (%)	<0.001	0.133	0.723
Total Lymphocytes (cells/μl)	<0.001	0.610	0.275
CD4+ T cell subsets (%)			
CD4+CD28-CD57-	0.047	0.685	0.403
CD4+PD1+	0.03	0.265	0.59
CD4+ T cell subsets (cells/μl)			
CD4+	<0.001	0.419	0.024
CD4+CD45RA+CCR7+	0.006	0.117	0.007
CD4+CD45RA-CCR7+	0.024	0.309	0.009
CD4+CD45RA+CD31+	<0.001	0.013	0.497
CD4+CD28+CD57-	<0.001	0.164	0.006
CD8+ T cell subsets (%)			
CD8+CD28+CD57-	0.004	<0.001	0.350
CD8+CD28-CD57-	0.001	0.358	0.242
CD8+ T cell subsets (cells/μl)			
CD8+	0.007	0.903	0.981
CD8+CD28+CD57-	<0.001	0.003	0.662
CD8+CD28-CD57-	0.037	0.640	0.990
B cell subsets (%)			
CD19+	<0.001	0.4	0.002
CD19+IGD+CD27+	0.047	0.109	0.034
B cell subsets (cells/μl)			
CD19+	<0.001	0.524	0.061
CD19+IGD+CD27+	<0.001	0.705	0.874
CD19+IGD+CD27-	<0.001	0.007	0.017
CD19+IGD-CD27+	<0.001	0.413	0.128
CD19+IGD-CD27-	<0.001	0.713	0.130

Supplemental Table 5. Differences in expression of CD28 and CD57 between patients on conventional hemodialysis (HD) and online hemodiafiltration (HDF).

	**HD**	**HDF**	***P***
**n**	36	26	
CD4+ subsets			
**CD4+CD28+CD57- (cells/µL)**	575(428-755)	682(482-820)	0.28
**CD4+CD28+CD57- (%)**	90.7(82.1-93.5)	94.9(89.3-97.0)	0.02
**CD4+CD28-CD57- (cells/µL)**	25(16-36)	16(9-25)	0.02
**CD4+CD28-CD57- (%)**	3.8(2.4-5.3)	2.1(1.3-3.3)	0.002
**CD4+CD28+CD57+ (cells/µL)**	4(2-11)	6(3-9)	0.41
**CD4+CD28+CD57+ (%)**	0.6(0.3-1.3)	0.7(0.3-1.5)	0.76
**CD4+CD28-CD57+ (cells/µL)**	21(5-47)	14(4-49)	0.34
**CD4+CD28-CD57+ (%)**	4.1(0.8-6.3)	2.4(0.4-7.8)	0.44
**CD4+CD28- (cells/µL)**	48(35-125)	32(18-74)	0.03
**CD4+CD28- (%)**	9.1(5.6-15.2)	4.1(2.4-9.2)	0.006
**CD4+CD57+ (cells/µL)**	26(9-55)	23(9-60)	0.71
**CD4+CD57+ (%)**	4.6(1.3-7.2)	3.4(0.8-7.4)	0.32
CD8+ subsets			
**CD8+CD28+CD57- (cells/µL)**	169(107-207)	150(113-192)	0.81
**CD8+CD28+CD57- (%)**	47.7(33.3-57.5)	47(30.9-71.5)	0.58
**CD8+CD28-CD57- (cells/µL)**	75(49-115)	46(33-92)	0.06
**CD8+CD28-CD57- (%)**	24.4(15.4-29.6)	14.2(10.2-23.4)	0.02
**CD8+CD28+CD57+ (cells/µL)**	6(3-14)	9(5-13)	0.21
**CD8+CD28+CD57+ (%)**	1.7(1.0-2.7)	2.2(1.5-3.5)	0.04
**CD8+CD28-CD57+ (cells/µL)**	90(32-189)	106(34-207)	0.81
**CD8+CD28-CD57+ (%)**	23.9(13.5-29.9)	30(12.9-46.1)	0.27
**CD8+CD28- (cells/µL)**	160(92-394)	142(76-322)	0.49
**CD8+CD28- (%)**	49.1(40.5-61.2)	51.9(24.8-66.5)	0.66
**CD8+CD57+ (cells/µL)**	97(38-186)	119(42-223)	0.50
**CD8+CD57+ (%)**	26.7(17.1-36.5)	32.3(15.6-48.9)	0.42

Supplemental Table 6. Conjugated monoclonal antibodies and combinations applied

**Tube 1**	**Tube 2**	**Tube 3**	**Tube 4**
T lymphocytes markers	*T lymphocytes markers*	*T lymphocytes markers*	*B lymphocytes markers*
CD45 PC7	CD45 PC7	CD45 PC7	CD45 PC7
CD3 FITC	CD3 PE	CD3 FITC	CD19 PC5
CD4 Pacific Blue	CD4 Pacific Blue	CD4 Pacific Blue	IgD FITC
CD8 PC5	CD8 PC5	CD8 PC5	CD27 ECD
CD45RA APC	CD45RA APC	CD45RA APC	
CCR7 PE	CD57 FITC	CD31 ECD	
CD28 ECD	CD28 ECD	PD1 PE	

Supplemental Table 7. T and B cell subsets as defined by the expression of surface markers

	**T lymphocytes markers (CD4+, CD8+)**	**B lymphocytes markers (CD19+)**
**Naive**	CD45RA+CCR7+	IgD+CD27-
**Recent Thymic Emigrants (RTE)**	CD45RA+CD31+	
**Central memory**	CD45RA-CCR7+	
**Effector memory**	CD45RA-CCR7-	
**TEMRA**	CD45RA+CCR7-	
**IgM memory**		IgD+CD27+
**Switched memory**		IgD-CD27+
**Late differentiated**	CD28-CD57+	IgD-CD27-
**Exhausted**	PD1+	

Supplemental Figure 1. ANCOVA for exhausted CD4+PD1+ T cells difference between healthy controls (N=34) and dialysis patients (N=62) according to age group. The difference in percentage of CD4+PD1+ T cells between patients and healthy individuals remained significant in multivariate analysis (p=0.003), independently of age (p=0.265).

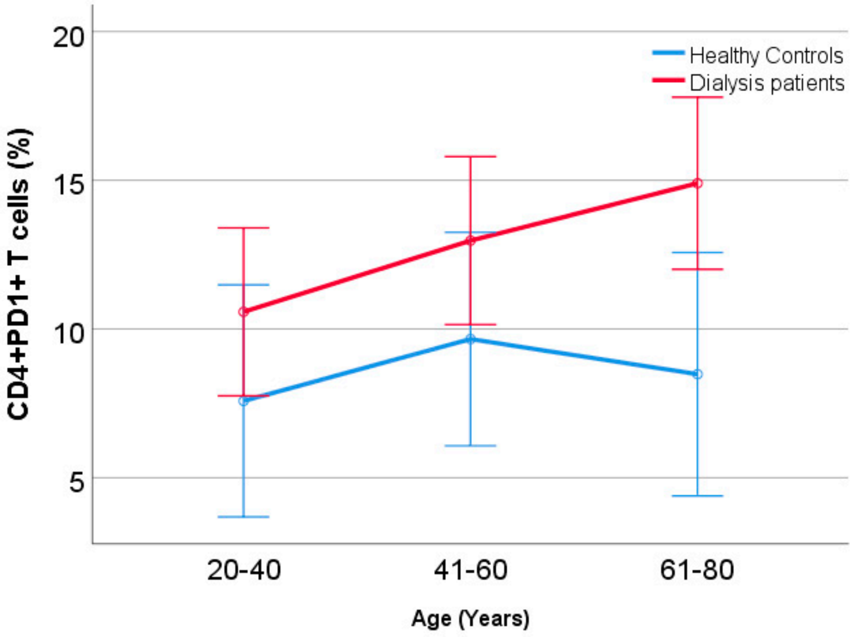

Supplemental Figure 2. Gating strategy. A. CD4+ T cells. B. CD8+ T cells. C. B cells

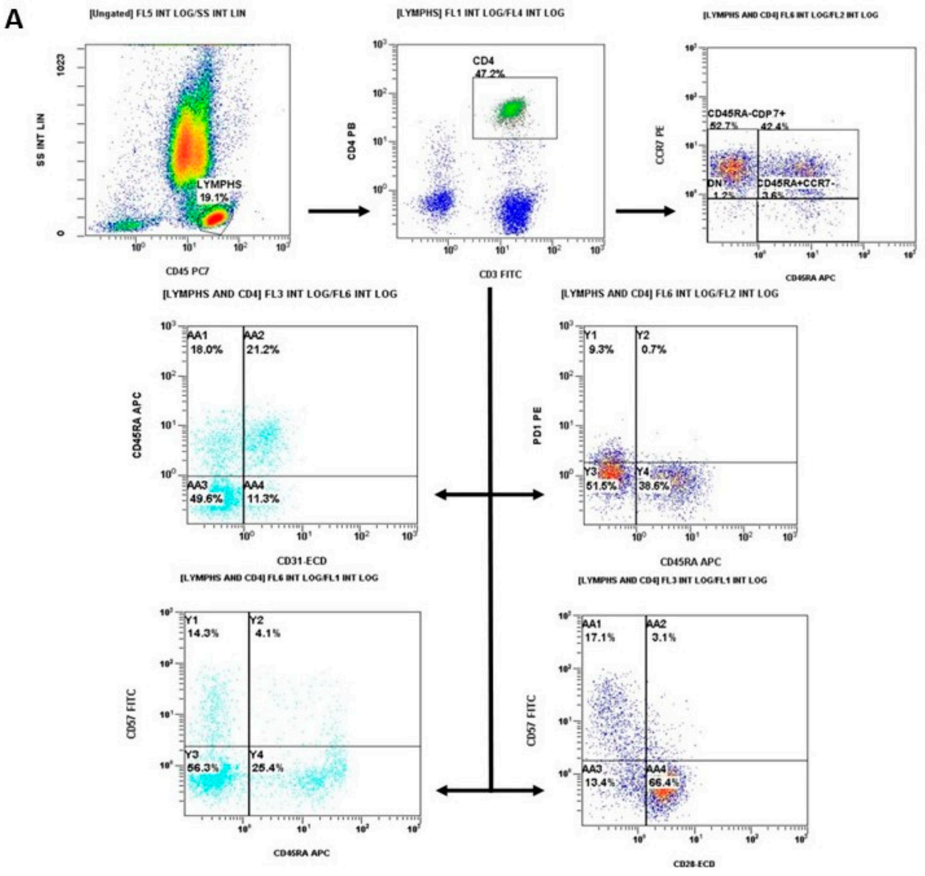

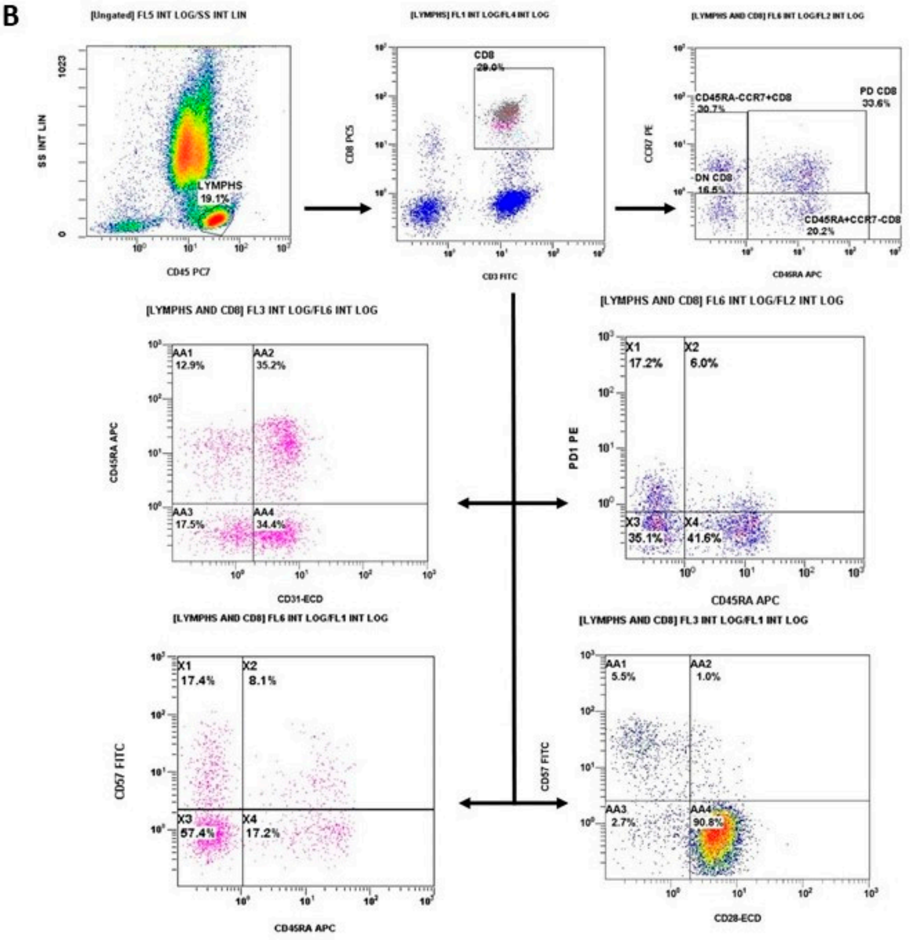

C

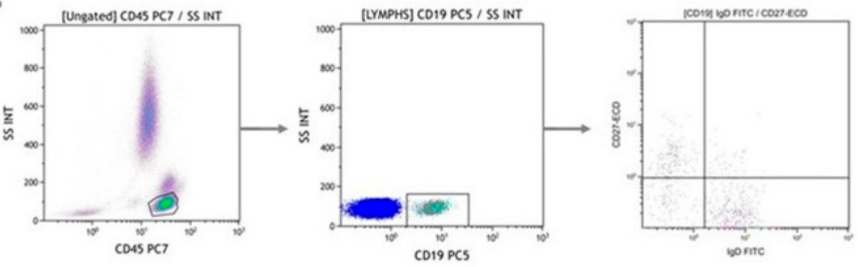
[Ungated] CD45 PC7 / SS INT
SS INT
CD45 PC7
[LYMPHS] CD19 PC5 / SS INT
SS INT
CD19 PC5
[CD19] IgD FITC / CD27-ECD
CD27-ECD
IgD FITC
